# Review of *"Fine Needle Aspiration Cytology of the Liver: Diagnostic algorithm a Southeast Asian Perspective" by A Wee and P Sampatankul*

**DOI:** 10.1186/1742-6413-2-10

**Published:** 2005-06-21

**Authors:** Isam A Eltoum

**Affiliations:** 1 Cytopathology Section, Division of Anatomic Pathology, University of Alabama at Birmingham, Birmingham, AL, USA

## 

Recently there has been an expansion of general and organ-specific cytopathology books. One may think there is no need for the latter since most of the general cytopathology books cover the cytopathology of organ sites extensively. This argument may not be true for all organ sites, particularly for the liver where there is a high demand for a book that illustrates the cytomorphologic aspects of liver lesions, especially in geographic regions such as Southeast Asia and Africa where liver disease is common. Wee and Sampatanukul's Fine Needle Aspiration of the Liver aim to fulfill the need for such a book.

This book presents a strong case for the complementary use of cytology and histology in the diagnostic work-up of liver mass and it is true to its title since it follows a simple diagnostic algorithm for the cytodiagnosis of hepatic lesions. As in clinical problem solving, this algorithm starts with the clinical findings followed by the imaging studies and then the cytologic findings. The algorithm ultimately allows a set of differential diagnoses from which a final diagnosis is arrived at through clinicopathological correlation and occasionally through the addition of ancillary studies such as immunohistochemistry. As such, the first part of the book is arranged in chapters each of which reflects a step in this process: chapter 2 describes clinical presentation, chapter 3 imaging, chapter 4 to 9 cytodiagnosis, chapter 10 ancillary testing and chapter 11 deals with clinicopathological correlation. The second part of the book presents "case studies" that beautifully illustrate this algorithm.

The book is well-written and well-illustrated. The authors presents a large collection of cases of primary and metastatic tumors of the liver as well as several other space occupying lesions of the liver that mimic primary liver cancers or their variants. They also review the diagnostic performance and accuracy of FNA as compared to needle biopsy and illustrate the pitfalls associated with cytodiagnosis of liver lesions. To demonstrate various microscopic features of these lesions, the authors use cytology-histology correlation extensively.

The book is moderate in size and is well-indexed, well-referenced and easy to read. The illustrations are high-quality color photographs with succinct informative legends. I wish the authors had included more cytologic rather than histologic illustrations of the various inclusions that are commonly seen in hepatocytes. This is also true for the immunohistochemistry.

During the three weeks that I had the book for review, I consulted it during the diagnostic workup for a case of hepatocellular carcinoma and again for a case of metastatic non-small cell carcinoma. Practicing pathologists as well as cytopathology trainees, especially those in the Southeast Asia and sub-Saharan Africa will find this book very appealing and a useful easy-to-use reference. Additionally, those of us who work in large centers where liver FNA is frequently performed will find this book indispensable.

